# Room Temperature Direct Electron Beam Lithography in a Condensed Copper Carboxylate

**DOI:** 10.3390/mi12050580

**Published:** 2021-05-20

**Authors:** Luisa Berger, Jakub Jurczyk, Katarzyna Madajska, Iwona B. Szymańska, Patrik Hoffmann, Ivo Utke

**Affiliations:** 1 Empa—Swiss Federal Laboratories for Materials Science and Technology, Laboratory for Mechanics of Materials and Nanostructures, Feuerwerkerstrasse 39, 3602 Thun, Switzerland; luisa.berger@epfl.ch (L.B.); jakub.jurczyk@empa.ch (J.J.); 2 Faculty of Physics and Applied Computer Science, AGH University of Science and Technology Krakow, Al. Mickiewicza 30, 30-059 Kraków, Poland; 3 Department of Chemistry, Nicolaus Copernicus University, Gagarina 7, 87-100 Toruń, Poland; 502533@doktorant.umk.pl (K.M.); pola@umk.pl (I.B.S.); 4 Empa—Swiss Federal Laboratories for Materials Science and Technology, Laboratory for Advanced Materials Processing, Feuerwerkerstrasse 39, 3602 Thun, Switzerland; patrik.hoffmann@empa.ch

**Keywords:** direct electron beam lithography, copper precursor, low-volatility precursor, direct-write

## Abstract

High-resolution metallic nanostructures can be fabricated with multistep processes, such as electron beam lithography or ice lithography. The gas-assisted direct-write technique known as focused electron beam induced deposition (FEBID) is more versatile than the other candidates. However, it suffers from low throughput. This work presents the combined approach of FEBID and the above-mentioned lithography techniques: direct electron beam lithography (D-EBL). A low-volatility copper precursor is locally condensed onto a room temperature substrate and acts as a positive tone resist. A focused electron beam then directly irradiates the desired patterns, leading to local molecule dissociation. By rinsing or sublimation, the non-irradiated precursor is removed, leaving copper-containing structures. Deposits were formed with drastically enhanced growth rates than FEBID, and their composition was found to be comparable to gas-assisted FEBID structures. The influence of electron scattering within the substrate as well as implementing a post-purification protocol were studied. The latter led to the agglomeration of high-purity copper crystals. We present this as a new approach to electron beam-induced fabrication of metallic nanostructures without the need for cryogenic or hot substrates. D-EBL promises fast and easy fabrication results.

## 1. Introduction

The fabrication of high-resolution metal structures in the micro- and nanometer scale is of great interest for both industry and research. Electron-beam lithography (EBL) is an exposure technique for fabricating micro- and nanostructures on an industrial scale. It is mainly used to manufacture 2D nanostructures with sub 200 nm feature sizes but can also reach the sub-10 nm range [1]. EBL is a multistep process consisting of the deposition and irradiation of a resist, the removal of the (non)exposed parts, the deposition of a metal layer and the final lift-off process leaving the intended structure on the substrate. EBL is commonly limited to flat surfaces (wafers) and 2D structures. Even though it is a multistep process, it can produce nanostructures with high throughput and excellent metal purity. In contrast, focused electron beam induced deposition (FEBID) allows the direct-write deposition of 2D and 3D structures on any substrate morphology [2]. However, it suffers from large amounts of deposit contamination and low growth rates limiting its use to prototyping and specialized academic use [3]. Direct-write deposition is achieved by supplying a metalorganic precursor locally to a substrate within the chamber of a scanning electron microscope (SEM). The focused electron beam of the SEM then irradiates the substrate with the desired pattern and electron dose. It leads to the dissociation of reversibly adsorbed precursor molecules. Non-volatile fragments form the deposit, while any volatile dissociation products are pumped away. This approach allowed the deposition of metal-containing quasi-1D nanodots [4,5,6], 2D structures [4,7,8] and complex 3D shapes [9,10,11,12,13,14,15] with a high lateral resolution. Ice lithography (IL) presents a technique where EBL and FEBID meet [16]. In IL, a precursor gas (e.g., water or alkanes) is supplied through a gas injection system (GIS), similar to the FEBID process, and condensed onto a cold substrate where it forms a homogeneous, amorphous ice film. Subsequently, the ice film is irradiated with an electron beam pattern, similar to EBL. In the case of water ice resists, the irradiated parts are removed (positive tone resist). The pattern is transferred onto the substrate via metal film deposition techniques. The desired metal structures are obtained after the lift-off process, or better called “melt-off”, by heating the substrate or immersing it into isopropyl alcohol at room temperature [17,18]. When using alkane ice, the irradiated pattern undergoes polymerization. The non-irradiated parts can be removed by heating the substrate (negative tone resist). The crosslinked carbon structures stay on the substrate and can, for instance, be used as a mask for plasma etching [19,20].

Unlike spin-coated resists used in EBL, the condensed ice films can also cover irregular, non-flat surfaces, extending the technique to complex substrates and already existing nanostructures [18]. Ultimately, IL was also shown to successfully deposit 3D nanostructures by layer stacking. In contrast to EBL, all irradiation and metallization steps in IL could be performed within the vacuum chamber of the SEM, reducing the fabrication process to a minimum [21].

A similar approach of resist-based lithography was performed with the common FEBID precursors MeCpPtMe_3_ and W(CO)_6_ as a resist [22,23,24,25]. For this, the precursor was delivered to the cold substrate with a GIS, forming a layer of precursor ice. Subsequent irradiation with the focused electron beam of the SEM (focused ion beam in the case of (WCO)_6_) led to the alteration of the precursor resist. Upon reheating the substrate, the non-irradiated precursor evaporated and left the irradiated patterns on the substrate. This technique represents a direct-write and resist-based deposition of metal–carbon structures with similar composition as the common room temperature FEBID of MeCpPtMe_3_ [22]. However, the growth rate of “cryo-FEBID” is over four orders of magnitude larger than RT-FEBID. Nevertheless, “cryo-FEBID” suffers from disadvantages, such as incorporating residual water or hydrocarbons from the SEM chamber into the condensate [22]. Similar to the 3D-IL, also “cryo-FEBID” could achieve 3D deposits by stacking multiple layers [23].

In all cases, the metal ion density in the condensed metalorganic resist concerning the metal density causes certain limitations for nanostructuring: For full decomposition to pure metal, the height of the original metalorganic layer film will shrink about 9 times for perfect two-dimensional decomposition [26]. If the metal atoms agglomerate within the metalorganic layer three-dimensionally, it will result in metal particles distributed in the remaining matrix before reaching the substrate at the full decomposition of the matrix.

The reports of metal–organic precursors being used as resists at room temperature (RT) are scattered over the past 35 years. Early electron beam exposure reports describe the deposition of gold with electrons from gold-containing inks [27]. Focused ion beams or electron beams deposited palladium from its spin-coated acetate films [28,29]. Iridium [30] and gold [31] structures were deposited using a focused ion beam. These were followed by multiple approaches of irradiating either passivated gold nanoparticles [32] or gold [33] and palladium [34] complexes with large organic thiolate ligands. For high metal purities, the latter had to undergo thermolysis to remove carbon residues. All resulted in high-resolution structures with features smaller than 30–100 nm. More recently, a different approach, using room-temperature ionic liquids, was reported for the formation of silver structures [35]. Even though the lateral resolution was not as high as other mentioned work, it resulted in high metal contents in the as-deposited state (<20 at % carbon).

This work aims to tie in with the previously reported results. Here we describe the fabrication of copper structures using the low-volatility precursor copper(II)pentafluoropropionate (Cu_2_(µ-O_2_CC_2_F_5_)_4_) (abbreviated as Cu_2_(pfp)_4_) on a room temperature substrate. A similar approach for the deposition of copper in a room temperature condensed precursor layer of copper acetylacetonate was performed with a UV light source [36]. Our work presents the first report of direct-write electron beam lithography (D-EBL) in a positive low-volatility copper precursor resist.

## 2. Materials and Methods

The precursor copper(II) pentafluoropropionate [Cu_2_(µ-O_2_CC_2_F_5_)_4_] (abbreviated as Cu_2_(pfp)_4_) was synthesized according to the literature [37].

The solid precursor Cu_2_(pfp)_4_ was filled in a gas injection system (GIS) made from chemically inert stainless steel. The GIS was equipped with a needle (0.38 mm ID) used to locally supply the precursor onto the substrate within the SEM chamber. Under vacuum, the GIS was heated to T_GIS_ = 140 °C for sufficient evaporation. After reaching the temperature, the needle opening was placed 200 µm above the substrate surface at the center of the sample. The precursor was condensed onto the RT substrate for 2.5 h. This process was repeated for all samples mentioned in this work.

The samples were irradiated with the focused electron beam of a Hitachi S3600 SEM equipped with a W-filament and a XENOS patterning engine with a beam blanker for beam control. The patterns were irradiated with primary energy of 25 keV and a beam current of 630 pA. After irradiation, the samples were developed in one of the following ways:

(A) Condensate dissolution in ethanol (EtOH): The samples were placed for 2 min into a beaker with clean EtOH. The resist was gently dissolved by swirling the beaker by hand. The sample was finally rinsed with fresh EtOH and then dried in air;
(B) Resist sublimation in high vacuum (HV): The samples were placed on a heatable stage and introduced into the SEM chamber. At a background pressure of 2 × 10^−5^ mbar, the stage was heated to 198 °C for 3.5 h. The temperature was measured both within the heating stage and on the substrate surface with Type-K thermocouples. The temperatures on the substrate surface did not exceed 125 °C. The maximum temperature was held for 35 min. Afterward, the heater was turned off, and the sample cooled down in HV.

All samples were analyzed with a high-resolution analytical SEM (Hitachi S4800). An EDAX silicon drift detector (SDD) was used for energy-dispersive X-ray spectroscopy (EDX). Spectra were recorded with an acceleration voltage of 3 keV, a beam current of 0.74 nA and a takeoff angle of 48° for 100 s. After subtracting the detector background signal, the k-ratios were determined using the standardless quantification of the EDAX Genesis software. The elemental composition of the deposits was determined with the thin-film analysis software SAMx STRATAGem [38,39] using the k-ratios. Any residual carbon and oxygen on the pristine substrate were considered by subtracting the measured quantities from the obtained deposit quantification.

The structure morphology was analyzed with atomic force microscopy (AFM) using an NT-MDT NTEGRA spectra system with Bruker antimony-doped silicon cantilevers. The data were treated with Gwyddion v2.42.

All data were treated further with Origin and Microsoft Excel. Figures were made with Adobe Illustrator CC or Affinity Designer (v1.8.6).

## 3. Results and Discussion

### 3.1. The Direct Electron Beam Lithography (D-EBL) Process in a Positive Low-Volatility Resist

“Cryo-FEBID” is a suitable technique for the deposition of metal-containing structures from metal–organic precursors that are volatile at room temperature. A similar approach can be used with low-volatility (LV) precursors, such as Cu_2_(pfp)_4_. The compound is a powder, stable at room temperature and evaporates only at elevated temperatures (T_GIS_ = 140 °C). For the deposition of the precursor resist, the substrate does not need to be cooled down to cryogenic temperatures but can be held at room temperature.

**Figure 1:**
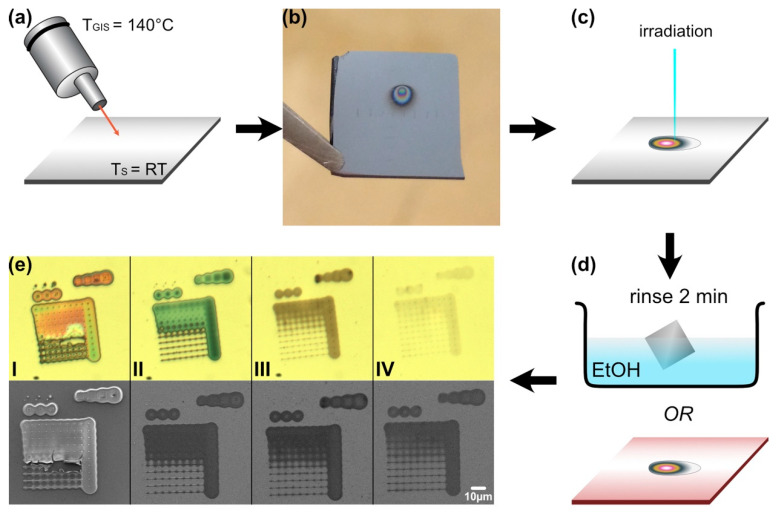
Experimental scheme of direct EBL in a Cu_2_(pfp)_4_ resist. (**a**) Precursor evaporation through GIS on a cold substrate. (**b**) Photo of the precursor condensate (“resist”). (**c**) Electron irradiation of the resist. (**d**) Pattern development by rinsing for 2 min in ethanol (EtOH) or heating the substrate for precursor sublimation. (**e**) Optical (upper) and SEM (lower) images of four irradiated structures after development in EtOH. The structures were written in differently thick condensate (gradient: center to outside = thick to thin = I to IV).

The “direct electron beam lithography” (D-EBL) process is illustrated in Figure 1. The resist film deposition was realized through evaporation of Cu_2_(pfp)_4_ through a GIS that was heated to 140 °C. The compound was condensed onto a silicon substrate with a native oxide layer (SiO_2_ (nat.)/Si), which was held at room temperature (Figure 1a). Figure 1b shows a photo of one substrate with Cu_2_(pfp)_4_ condensate. It is visible as a pear-shaped condensate in all refractive colors, which occurred due to a thickness gradient. This thickness gradient corresponds to the variation in molecule flux impinging on the substrate surface (S1, Supporting Information). The condensate thickness decreased from the center to the outside.

After forming the resist film, a defined pattern was irradiated. The detailed irradiation parameters are listed in S2 of the Supporting Information. For each sample, the pattern was repeated in four different regions of the condensate to obtain irradiation at four different resist thicknesses (Figure 1c). The pattern consisted of squares and dot arrays of varying electron doses. The dots were deposited in an array of 10 dots per line with a distance of 5 µm, written from left to right and top–down. The arrays were deposited with an inherent bug within the XENOS lithography software. The last object of an array is irradiated much longer than programmed. Therefore, any irregularity in the last row originates in this bug that could not be solved by the software provider so far. All other dots, however, were deposited with the dwell times indicated in S2. All structures were written according to their numeration (i.e., starting with 1 and ending with 8_4).

After exposure, all non-irradiated condensate was removed for pattern development either by dissolution in ethanol (EtOH) or by evaporation in the SEM chamber by heating the substrate (Figure 1d). Optical and SEM overview images of the pattern after EtOH development are shown in Figure 1e with images of the optical microscope in the upper and the HR-SEM in the lower row. The numbers I to IV correspond to the position of irradiation in the condensate, with I being in the thickest, central area and IV in the thinnest, outmost area of the resist. The different appearances of the structures can be observed in these images and are consistent with the condensate thickness. Thin deposits appear faint (i.e., Figure 1e(IV)), thick deposits show a strong contrast and even interference colors (i.e., Figure 1e(I)).

### 3.2. Development in EtOH

**Figure 2:**
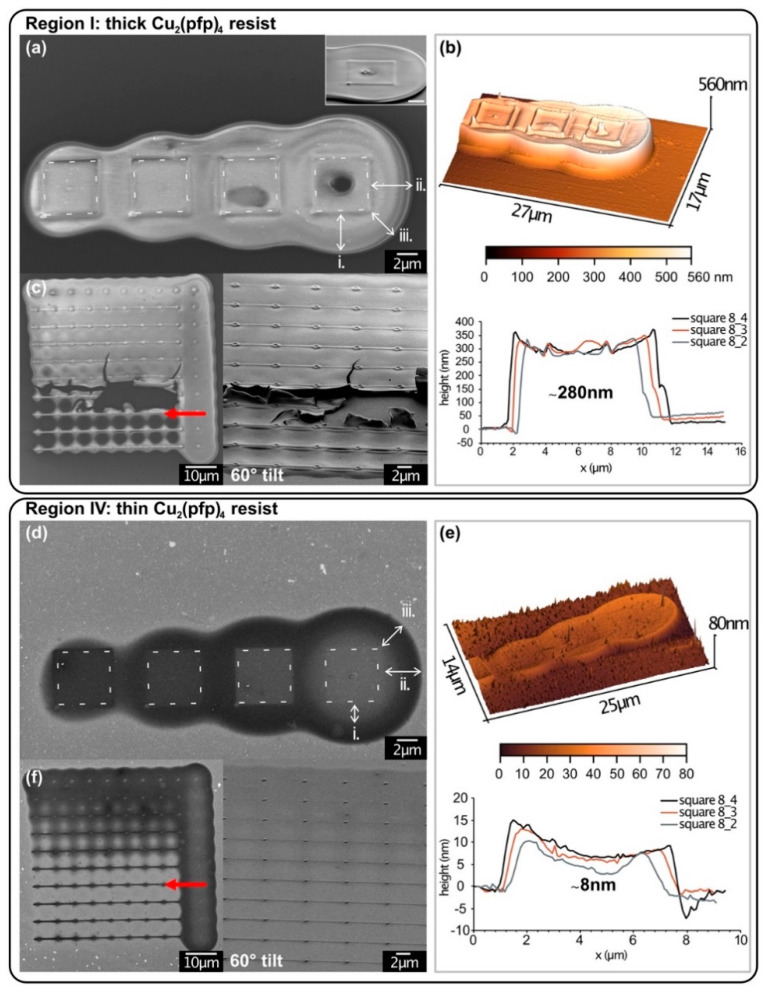
D-EBL structures from Cu_2_(pfp)_4_ on a SiO_2_(nat.)/Si substrate after development in EtOH. The shown structures are 5 × 5 µm^2^ squares and a dot array with varied e-doses. (**a**) SEM images of four squares deposited with increasing e-dose (left to right) in thick condensate (I). Inset: tilted view of parts of the structure. (**b**) 3D AFM image and height profiles of the squares; the average structure thickness marked in the graph. (**c**) SEM images of the dot arrays in the thick resist region (I). The last (right) dot in each line is a long-time exposure of 30 ms. Right: zoom and tilted view. Delamination of the irradiated condensate is visible. The red arrow indicates the e-dose threshold (see text). (**d**–**f**) Analogous images of the structures in the thin resist area (IV). Strongly discernible halos are observed for all structures. The halo sizes i, ii and iii marked in (**a**,**d**) are quantified in Table 1.

As described above, the pattern was repeatedly deposited in different regions of the condensate to study any changes. Figure 2 details the structures 8_1-4 and the dot array 7_1-10 of the thickest and thinnest region after development in EtOH, as seen in Figure 1e(I,IV). The original pattern of the 5 × 5 µm^2^ squares in Figure 2a,d is indicated by white dashed lines to guide the eye. It is clearly visible that all squares feature a halo around the squares, originating from backscattered electrons (BSE) escaping the Si bulk substrate up to a certain radius r_BSE,Si_, which depends on the beam energy. In the case of 25 keV primary energy, as used in this experiment, BSE escapes until a theoretically calculated radius of 3.9 µm [40]. For both regions, the halo surrounding square 8_4 was measured in the SEM images. Three different distances were measured (i) from the square side to the bright halo edge, (ii) the dark halo edge and (iii) from the square corner to the dark halo edge. The values are summarized in Table 1.

An increasing halo diameter was observed with increasing electron dose from left to right. This is linked to the increasing number of BSE being emitted at large radii with increasing electron dose. In these experiments, the dose was varied via dwell time variation. Therefore, the square with the smallest electron dose was deposited with the shortest dwell times at constant electron flux and caused a smaller number of electrons to scatter within the bulk. This also means that the electron dose of BSE was significantly smaller than for long dwell times and insufficient to cause electron-induced chemistry within the condensate.

For the thick deposit, the radius diverges ±10% from the theoretical value, with the lateral distance (ii side—dark halo edge) being larger and the second lateral (i side—bright edge) and diagonal distance (iii corner—halo edge) being smaller than the theoretical distance (Table 1). The halo of the thinner deposit diverged largely from the theoretical exit radius for distances (i) and (iii). At the lateral distance (ii), however, it shows good agreement. Therefore, the bright halo edge in the thin deposit corresponds to a threshold where “deposition” changes and is visible as contrast change at a shorter range, which could correspond to an electron dose threshold, which was necessary for precursor dissociation. The same trend was visible in the dot arrays, where the electron dose per dot decreases from top to bottom. A threshold for sufficient electron dose, which then caused a halo deposit or not, could be observed in the dot arrays. The rows are indicated with red arrows in Figure 2c,f.

The observed discrepancy between the two resist regions I and IV could arise from additional electron scattering within the thick condensate, increasing the halo radius. In addition, electron-induced dissociation of the condensate could have led to forming volatile fragments, which could not desorb from within the resist. This led to a bulging of the resist material, increasing its size and visible halo diameter. In contrast, most BSE interacting with the thin condensate were scattered within the bulk substrate.

Since the condensate thickness could not be determined beforehand, AFM measurements of the structures were performed after development. The results are shown in Figure 2b,e. The graphs depict three profiles over the squares 8_2-4 (c.f. Supplementary Materials S2). The average structure thickness was determined to be 280 ± 30 nm and 8 ± 3 nm, respectively, showing a direct dependency of the condensate film thickness with the structure thickness. This corresponds to a deposition rate of 1.26 nm/s in the thick deposit, which is about 4 orders of magnitudes larger than a comparable gas-assisted FEBID square (<0.03 nm/s). A similar enhancement of the deposition rate was reported for “cryo-FEBID” experiments [23].

It may seem surprising that the halo thickness does not decrease towards the outside of the structure. Intuitively, it would be expected to become gradually thinner as the number of BSE decreases with increasing distance from the beam center, as shown by Monte Carlo simulations [41]. However, this is not the case here. The abrupt end and constant thickness of the halo lead to the following considerations:

1. After a certain radius, r(halo), the electron dose is too low to form a deposit. Any dissociation formed at a smaller electron dose beyond r(halo) led to insufficient decomposition of the precursor molecules;
2. These insufficiently decomposed molecules were not forming stable deposits so that they were easily removed in the development step of the process (i.e., rinsed off in EtOH or evaporated in HV);
3. A thickness gradient depending on the number of BSE cannot be observed because the deposit thickness depends on the thickness of the condensed film. Any deposit thinner than that seems to be removed in the development step (see point 2).

Additionally, the AFM image in Figure 2b exhibits three characteristics: a 3D dot on the lower-left corner, a 3D dot in the center and a “wall” at the outside edge of each square. The squares were deposited with a spiral writing technique starting from the lower-left corner and spiraling inwards. The lithography software dwells longer on the starting point of the structure, leading to the dot on the corner. When spiraling the pattern inwards, more and more of the beam overlaps and accumulates a higher effective number of electrons in the center, increasing the deposition rate locally. When irradiating a resist, however, no new molecules diffuse towards the irradiation site. Therefore, an increase in deposition rate is not possible in the classic sense of FEBID growth. This means that either molecule from the surrounding condensate diffused towards the high irradiation points (corner/center), which seems unlikely for an LV precursor at RT. Or the accumulation of electrons in these regions led to charge accumulation within the thick, nonconductive resist and caused it to bulge. Since the AFM and HR-SEM images were taken after EDX measurements, where small areas of the structures were probed with a high beam current, the latter seems a more probable explanation. The dark areas in squares 8_3 and 8_4 in Figure 2a correspond to the regions of EDX measurement. They are also visible in the 3D AFM image, especially in square 8_3, where the dark area can be identified as a bulge. These features are not visible in the thin condensate structures. They can, therefore, be attributed to the thick resist, which trapped volatile fragments. Similarly, it was reported that during electron-induced irradiation of copper(II) oxalate, physisorbed CO_2_, which was formed upon decomposition, was trapped within the film [42,43].

Figure 2c depicts the dot array irradiated in the thick condensate. Here it should be stressed that the deposit is delaminated from the substrate upon development. It is especially apparent in the tilt view image on the right. The delamination could arise from stresses within the thick deposit upon development in EtOH. This is a common challenge in standard EBL and photolithography.

Another notable difference between the thick and thin deposits is the inversed contrast in the SEM images. While the thick structures appear bright compared to the substrate, the thin deposits are darker than the surrounding background. The bright contrast in Figure 2a,c could be attributed to charging effects within the less conductive resist material rather than a high metal content. In contrast, the dark contrast in Figure 2d,f might have originated from carbonaceous material in the halo and the slightly brighter contrast within the deposition area/center from a higher metal content. As shown in the AFM measurements (Figure 2e), the deposit thickness is uniform over the halo and square deposit, meaning that any charging effects should be visible as in Figure 2a.

### 3.3. Development via Annealing

**Figure 3:**
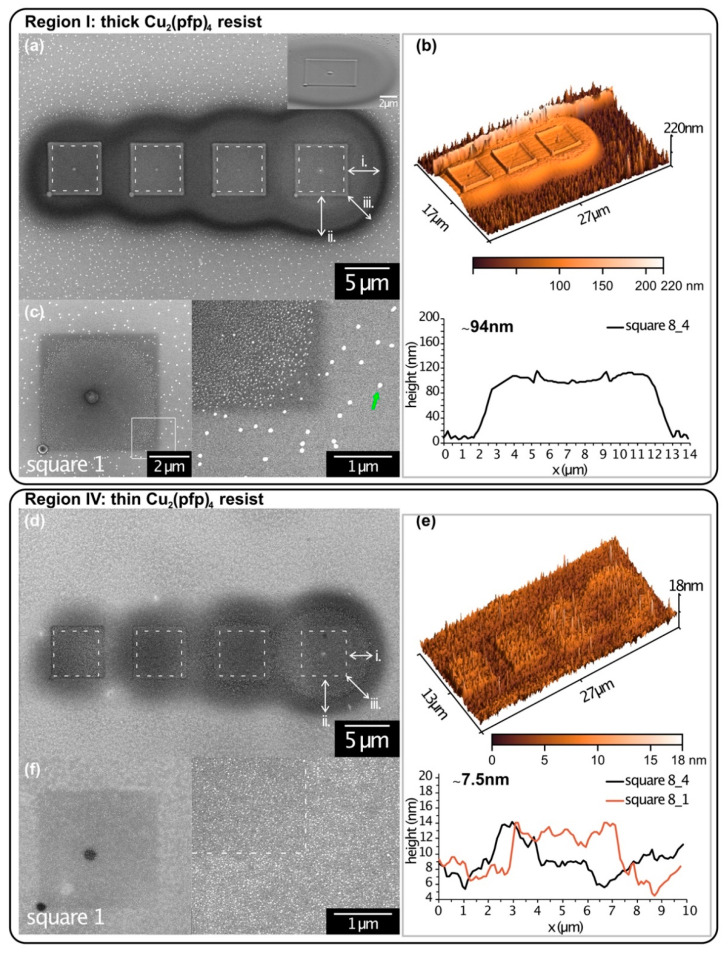
D-EBL structures from Cu_2_(pfp)_4_ on a SiO_2_ (nat.)/Si substrate after development via thermal resist evaporation in HV (substrate heated to T_S_ = 190 °C). (**a**) SEM image of the structures irradiated in the thick resist area (I). Halo radii are indicated. The inset shows a tilted view of square 8_4. The green arrow indicates a crystallite in the non-irradiated region. (**b**) 3D AFM image and height profile of these structures. (**c**) SEM images of square 1 (c.f. Supplementary Materials S2) with a high magnification image of the lower-left corner. (**d**–**f**) Results of the same structures in the thinnest region of the condensate (IV).

Other samples were prepared with the same precursor condensation protocol but a different development process. The structures were developed by annealing the substrate to T_S_ > 140 °C in a high vacuum (HV, 2 × 10^−5^ mbar base pressure). As apparent from the AFM measurements in Figure 3, they are significantly thinner than the corresponding EtOH developed structures. The annealing probably caused the evaporation of dissociated fragments in the structure, which were encapsulated and trapped in the EtOH sample. This might have led to the shrinkage of the deposit. However, it should also be noted that the thickness of the condensate could not be determined beforehand. The actual condensate thickness at the area of irradiation was, therefore, not compared between both samples. Since the condensation step was identical for both samples, it was assumed that all samples have a similar condensate layer. Analogous to the sample developed in EtOH (Figure 2), the structures 8_1-4 are shown in Figure 3, with (a) in the thickest region (I) and (d) in the thinnest region (IV) of the condensate. Corresponding AFM images and line profiles for size determination are shown alongside (Figure 3b,e). Additionally, Figure 3c,f depict SEM images of another square deposit (square 1, c.f. Supplementary Materials S2) in the respective region. The image on the right depicts a high-magnification image of the lower right corner of each deposit.

The SEM and AFM images demonstrate a similar appearance compared to the EtOH sample, including the halo around the squares. This was not surprising, as the primary beam energy and substrate density did not change. Therefore, a theoretical exit radius of r_BSE,Si_ = 3.9 µm was expected. The halo radii indicated were measured from the SEM images and are noted in Table 1. The values lie within the same range as for the EtOH developed sample. The thicker deposits show good agreement with 5–10% deviations, and thin deposits show similar deviations as above. Only the bright halo edge (i) is slightly larger than for the EtOH developed sample but is still significantly smaller than r_BSE,Si_.

The most prominent difference to the EtOH developed sample is the presence of bright crystallites in the background of the sample (green arrow in Figure 3c). In areas where more Cu_2_(pfp)_4_ was available, i.e., thicker condensate, the particles are larger and scattered further apart. In the thin condensate regions, smaller clusters formed are more numerous and packed more closely. In square 1 of region I (Figure 3c), forming locally concentrated crystallites was very visible. Their size is clearly distinguishable from the other crystals (green arrow) and is more concentrated towards the square’s center. They can, therefore, be ascribed to the electron-induced dissociation process. The decreasing number of crystallites towards the corners indicates an electron dose, which is insufficient for uniform deposition. In the future, this could be used for the determination of the minimum electron dose necessary for the complete dissociation of the film before development. Square 1 in region IV, though (Figure 3d), is barely distinguishable from the background. A white line was inserted to guide the eye in the high magnification image. Apart from a slightly darker shade, the irradiated area does not look different from the thermally dissociated precursor outside the patterned region. The darker contrast within the structure most probably originates from additional carbon deposition through electron-induced dissociation.

### 3.4. Chemical Analysis of the Developed Structures

**Figure 4:**
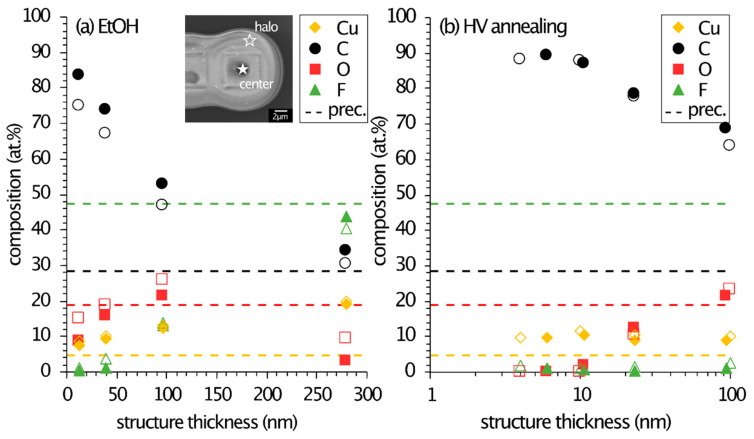
EDX quantification results of the D-EBL structures developed (**a**) in EtOH and (**b**) by annealing HV. The EDX spectra were recorded locally with 3 keV within the structure 8_4 (filled symbols) and in its respective halo (empty symbols). The position of the spectra collection is noted in the inset. Each structure thickness was determined with AFM and correspond to regions I–IV as in Figure 1e. The pristine precursor values are noted for comparison as dashed lines. Note: *x*-axis in (**b**) is a logarithmic scale. Atomic concentrations have an uncertainty of ±5 at % (not noted in this graph).

To determine the metal contents within the D-EBL deposits after development, EDX measurements were locally conducted on square 8_4 in each region, both within the structures (center) and in the respective halo. The quantification results of each irradiation field in varying condensate thickness are illustrated in Figure 4 and are background subtracted and thin-film corrected. The values measured in the center (filled symbols) and halo (empty symbols) are noted separately. The pristine precursor composition is noted alongside for comparison (dashed lines). The structure thickness was determined with AFM measurements (c.f. Figure 2c,d) and increased from the fields IV to I. It should be noted that in the case of EtOH development, the background signals were clean, and only small amounts of carbon and oxygen <2 at % were detected, which was attributed to background contamination during the EDX measurements. Therefore, the development process in EtOH was successful, removing any non-irradiated Cu_2_(pfp)_4_ residuals.

The EDX quantification in Figure 4a shows that there was little to no difference in the composition between the center and halo regions. All fluctuation lies within the measurement uncertainty of ±5 at %. However, there is a large variation within the differently thick structures, i.e., regions. With increasing thickness, the carbon concentration decreased, and the fluorine content increased. While the oxygen content increased with larger thickness, it abruptly dropped to <10 at % for the thickest structure. All values converged towards the original precursor composition but with decreased oxygen and increased copper content (up to 20 at %). For very thin deposits, the carbon content increased drastically.

The convergence towards precursor composition could be explained by the almost bulk dimension of the resist in the region I. The resulting structures were about 280 nm thick, and no more substrate signal was detected at 3 keV acceleration voltage. It is possible that electron irradiation polymerized the top layers of the resist, therefore encapsulating the precursor and preventing the desorption of electron-induced fluorinated fragments. Therefore, the composition within the bulk did not change significantly from the original composition, except for oxygen. This could be due to CO_2_ desorption. As the oxygen content decreased, the copper content increased simultaneously. The bulky matrix could have acted as an oxidation barrier, preventing the oxidation of any formed copper particles in this structure, also causing a lower oxygen content than the thinner deposits.

The lack of an oxidation barrier might also be the reason why thinner deposits have lower copper and higher oxygen concentrations. Copper–oxygen ratios reached Cu:O = 1:1–1.5, which strongly indicates forming CuO, as reported for common gas-assisted FEBID structures from Cu_2_(pfp)_4_ [44]. The low fluorine values indicate the electron-induced desorption of fluorine-containing species.

The EDX quantification results for the annealed structure showed similar trends for each element (Figure 4b). The thickest structure in the region I, however, did not reach pristine precursor values. This could be assigned to the different development processes. The irradiated structures were annealed to high temperatures, causing an additional alteration of the structures.

**Figure 5:**
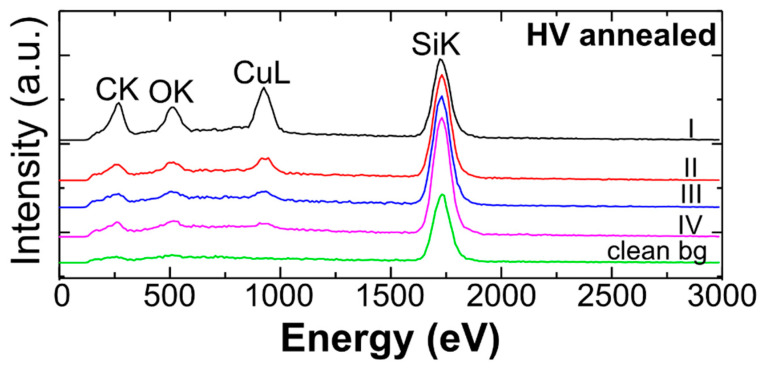
EDX spectra of non-irradiated areas in proximity to irradiation fields I–IV (HV annealed sample). Green spectrum: clean SiO_2_ (nat.)/Si surface was measured outside the condensate area for comparison.

The EDX background measurement illustrated in Figure 5 clearly shows the presence of copper and more carbon and oxygen within the different regions of the condensate (I–IV). In comparison, no copper and very weak signals of carbon and oxygen were detected on the clean SiO_2_ (nat.)/Si surface. Therefore, the crystallites outside the irradiated patterns are copper-containing clusters, which formed upon annealing. The development process probably induced thermal decomposition of the precursor on the entire substrate. Due to its high mobility, copper agglomerated into these larger features. To achieve a clean development process, without thermal decomposition and codeposition, lower annealing temperatures could lead to better results.

As the metal content is slightly lower for these D-EBL structures compared to as-deposited FEBID structures, we expect the electrical resistivity to be similar or higher than reported previously [44]. Electrical characterizations were not performed with these structures.

### 3.5. Post-Purification of D-EBL Structures

**Figure 6:**
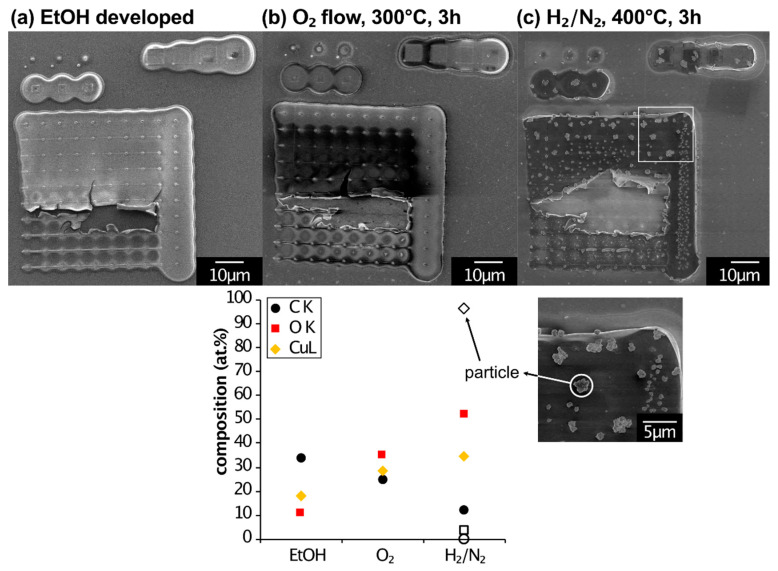
Post-purification of D-EBL structures in region I. (**a**) The as-deposited structure after development in EtOH. (**b**) The oxidized structure after 3 h in oxygen flow at 300 °C. (**c**) The reduced structure after 3 h in form gas at 400 °C. Large clusters have formed (see high magnification region below). The graph shows the quantification results of local EDX measurements at each processing step. The empty black data points refer to the local EDX measurement of a large cluster (see high magnification image). It consists of pure copper (96 at %).

Following the 2-step purification protocol described for this precursor in our earlier work [44], the D-EBL structures were annealed subsequently in the reactive gases O_2_ and H_2_/N_2_. The result of the region I (thickest condensate) after EtOH development is summarized in Figure 6. The SEM images show the whole irradiation pattern (a) after development, (b) after oxidation at 300 °C for 3 h and (c) after reduction in form gas at 400 °C for 3 h. The graph shows the quantification results of local EDX measurements taken in square 8_4 (as above) at each processing step. While the appearance from (a) to (b) did not change significantly, the elemental composition changed notably. Further processing in form gas changed the appearance drastically. Large clusters precipitated on the irradiated areas. The EDX measurement at the common region (square 8_4) showed a further decrease in carbon content, increased copper content (35 at %) and increased oxygen content, even though it was annealed in a reducing atmosphere. Additionally, the precipitates were locally measured and analyzed with EDX. These are shown as the empty symbols in the graph. They turned out to be pure copper clusters (96 at %), similar to what was reported for the purification of regular FEBI deposits from Cu_2_(pfp)_4_ [44].

The purification of copper-containing D-EBL structures resulted in the precipitation of pure copper crystals and clusters. The other regions were also analyzed but did not give valuable results. The high annealing temperatures may have led to the diffusion of copper into the silicon substrate, falsifying the quantification results. The native oxide layer on the Si substrate used for this experiment was not an effective diffusion barrier. Therefore, future experiments should be conducted on different substrates with a diffusion barrier (i.e., >200 nm SiO_2_, SiN_x_, AlN, etc.).

## 4. Conclusions

This work presents an alternative deposition approach for the direct-write fabrication of metallic structures using a focused electron beam. Based on the common EBL process and more recent modifications, known as ice lithography and “cryo-FEBID”, the low-volatility compound Cu_2_(pfp)_4_ was used as a lithography resist. Since this approach needed neither an additional metal deposition step nor cryogenic temperatures for the resist, it was referred to as “direct-write electron-beam lithography” (D-EBL). In general, the D-EBL process has shown to be suitable for the deposition of low-volatility precursors and the fabrication of structures within the condensate. It exhibited some advantages over the “classic” gas-assisted FEBID approach:

1. The substrate was held at room temperature during deposition so that no thermal drift occurred. This is usually an issue of LV precursor FEBID, where the substrate must be heated to elevated temperatures (e.g., Cu_2_(pfp)_4_: T_S_ = 135 °C).
2. The deposition rate was up to about 4 orders of magnitude larger than for the gas-assisted process. In contrast to “cryo-FEBID”, this RT approach has the advantage that no water or other contaminants present in the chamber were condensed with the precursor.
3. This process allows a higher processing throughput. After preparing the substrates, multiple samples can be irradiated on regular SEM stages. A larger number of patterns are possible since irradiation times can be reduced drastically.

The D-EBL structures featured metal contents comparable to gas-assisted FEBID structures from Cu_2_(pfp)_4_. The resist’s sensitivity towards BSE halo deposition posed another challenge. It should be easily avoided by adjusting the electron dose. Lastly, the thickness and uniformity of the condensate could not yet be controlled. Surely, by exploring the deposition and developing parameters further, this technique presents a promising approach for fabricating metal-nanostructures.

## Figures and Tables

**Table 1:** Halo radii as measured from squares 8_4 in Figure 2 and Figure 3. The value r_BSE,Si_(25 keV), denotes the maximum exit range of backscattered electrons on a thick Si substrate.

Halo Radius *^a^*	EtOH		HV Annealing		
	(a)	(b)	(a)	(b)	r_BSE,Si_ (25 keV)
i Side—bright edge	3.6 µm	1.8 µm	3.7 µm	2.6 µm	3.9 µm
ii Side—dark edge	4.3 µm	3.9 µm	4.5 µm	3.6 µm	
iii Corner—dark edge	3.5 µm	2.9 µm	3.7 µm	3.4 µm	

*^a^* Distances measured by eye in SEM images. Measurement uncertainty is ± 0.05 µm.

## Supplementary Materials

The following are available online at https://www.mdpi.com/article/10.3390/mi12050580/s1, Figure S1: Impinging Precursor Flux Simulation, S2: Irradiation Pattern and Detailed Parameters (with Figure S2: Irradiation pattern with structure numbers and Table S3: Irradiation details of the D-EBL pattern).
