# Supplementary material for: Room Temperature Direct Electron Beam Lithography in a Condensed Copper Carboxylate
Source: Micromachines (Basel). 2021 May 20;12(5):580. doi: 10.3390/mi12050580 (PMC8161174; doi:10.3390/mi12050580)
Supplement: Supplementary file 1 [file micromachines-12-00580-s001.zip › micromachines-1148420-supplementary.pdf]

Supporting Information to the Mansucript

Room Temperature Direct Electron Beam Lithography in a Condensed Copper Carboxylate

by Luisa Berger, Jakub Jurczyk, Katarzyna Madajska, Iwona B. Szymańska, Patrik Hoffmann and Ivo Utke

S1 – Impinging Precursor Flux Simulation

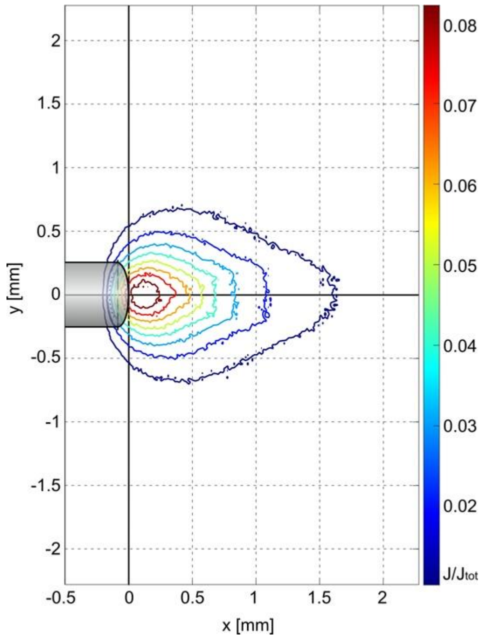

Figure S1 Simulation of the impinging molecule flux exiting from the nozzle.

Precursor flux simulation arriving on the substrate from a GIS nozzle with 380 µm inner diameter and positioned 200 µm over the substrate surface. The color scheme represents the fraction of molecule flux J/J_{tot} arriving on the xy-position on the substrate surface in respect to the total molecule flux J_{tot}. J_{tot} was determined experimentally using the precursor mass loss measured after the experiment.

The simulation was done using the GIS Simulator [1] with the following simulation parameters: 10^7 molecules, 256 grid points, uptake coefficient (in nozzle) = 0, needle length l = 4mm, nozzle angle α = 70 °, height above substrate h = 200 µm.

S2 – Irradiation Pattern and Detailed Parameters

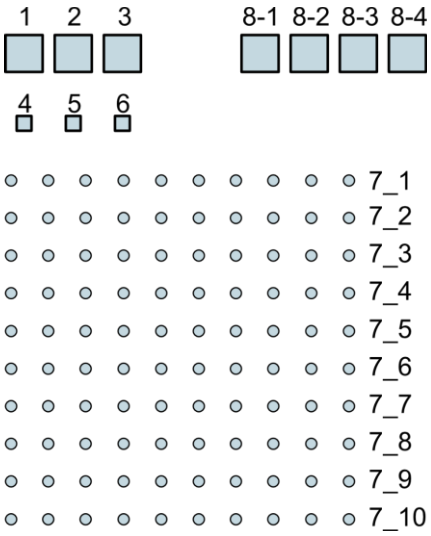

Figure S2 Irradiation pattern with structure numbers. Detailed irradiation parameters are listed in Table S-3.

Table S3 Irradiation details of the D-EBL pattern.

	size (μm x μm)	t_d (ns)	repetitions	step size (nm)	e-dose (μC/cm^2)
1	5x5	3720	125		
2	5x5	1860	250	150	$3.26 \cdot 10^{-4}$
3	5x5	1120	416		
4	2x2	1000	125		
5	2x2	500	250	3	$3.50 \cdot 10^{-4}$
6	2x2	200	416		
7_1		50000			$3.09 \cdot 10^{-2}$
7_2		10000			$6.19 \cdot 10^{-3}$
7_3		7500			$4.65 \cdot 10^{-3}$
7_4		5000			$3.09 \cdot 10^{-3}$
7_5	dot	4000	100000	5000	$2.48 \cdot 10^{-3}$
7_6		2000			$1.24 \cdot 10^{-3}$
7_7		1000			$6.19 \cdot 10^{-4}$
7_8		750			$4.65 \cdot 10^{-4}$
7_9		500			$3.09 \cdot 10^{-4}$
7_10		200			$1.24 \cdot 10^{-4}$
8_1	5x5	500			$1.40 \cdot 10^{-2}$
8_2	5x5	1000	2500	37.5	$2.80 \cdot 10^{-2}$
8_3	5x5	2500			$7.00 \cdot 10^{-2}$
8_4	5x5	5000			$1.40 \cdot 10^{-2}$

1. Friedli, V.; Utke, I. Optimized molecule supply from nozzle-based gas injection systems for focused electron- and ion-beam induced deposition and etching: simulation and experiment. *J. Phys. D. Appl. Phys.* **2009**, *42*, 125305, doi:10.1088/0022-3727/42/12/125305.
